# Cancer mortality in the West Bank, Occupied Palestinian Territory

**DOI:** 10.1186/s12889-016-2715-8

**Published:** 2016-01-26

**Authors:** Niveen M. E. Abu-Rmeileh, Emilio Antonio Luca Gianicolo, Antonella Bruni, Suzan Mitwali, Maurizio Portaluri, Jawad Bitar, Mutaem Hamad, Rita Giacaman, Maria Angela Vigotti

**Affiliations:** 1 Institute of Community and Public Health, Birzeit Univeristy, Ramallah, Occupied Palestinian Territory; 2 Institute of Clinical Physiology of the National Research Council, Lecce, Italy; 3 Institute of Medical Biostatistics, Epidemiology and Informatics, Johannes Gutenberg - Universität, Mainz, Germany; 4 Radiotherapy Department “Perrino” General Hospital, Brindisi, Italy; 5 Health Information Management Centre, Ministry of Health, Nablus, Occupied Palestinian Territory; 6 Biology Department, University of Pisa, Pisa, Italy; 7 Institute of Clinical Physiology of the National Research Council, Lecce, Pisa, Italy

## Abstract

**Background:**

The burden of cancer is difficult to study in the context of the occupied Palestinian territory because of the limited data available. This study aims to evaluate the quality of mortality data and to investigate cancer mortality patterns in the occupied Palestinian territory’s West Bank governorates from 1999 to 2009.

**Methods:**

Death certificates collected by the Palestinian Ministry of Health for Palestinians living in the West Bank were used. Direct and indirect age-standardised mortality rates were computed and used to compare different governorates according to total and specific cancer mortality. Furthermore, standardised proportional mortality ratios were calculated to compare mortality by urban, rural and camp locales.

**Results:**

The most common cause of death out of all cancer types was lung cancer among males (22.8 %) and breast cancer among females (21.5 %) followed by prostate cancer for males (9.5 %) and by colon cancer for females (11.4 %). Regional variations in cancer-specific causes of death were observed. The central- West Bank governorates had the lowest mortality for most cancer types among men and women. Mortality for lung cancer was highest in the north among men (SMR 109.6; 95%CI 99.5-120.4). For prostate cancer, mortality was highest in the north (SMR 103.6; 95%CI 88.5-120.5) and in the south (SMR 118.6; 95%CI 98.9-141.0). Breast cancer mortality was highest in the south (SMR 119.3; 95%CI 103.9-136.2). Similar mortality rate patterns were found in urban, rural and camp locales.

**Conclusion:**

The quality of the Palestinian mortality registry has improved over time. Results in the West Bank governorates present different mortality patterns. The differences might be explained by personal, contextual and environmental factors that need future in-depth investigations.

**Electronic supplementary material:**

The online version of this article (doi:10.1186/s12889-016-2715-8) contains supplementary material, which is available to authorized users.

## Background

The burden of cancer is increasing worldwide with 12.7 million new cancer cases yearly and 7.6 million cancer deaths occurring in 2008 according to GLOBOCAN series [1]. It is predicted that there will be more than 15 million new cancer cases and 12 million deaths due to cancer in 2020 worldwide [2].

**Fig. 1:**
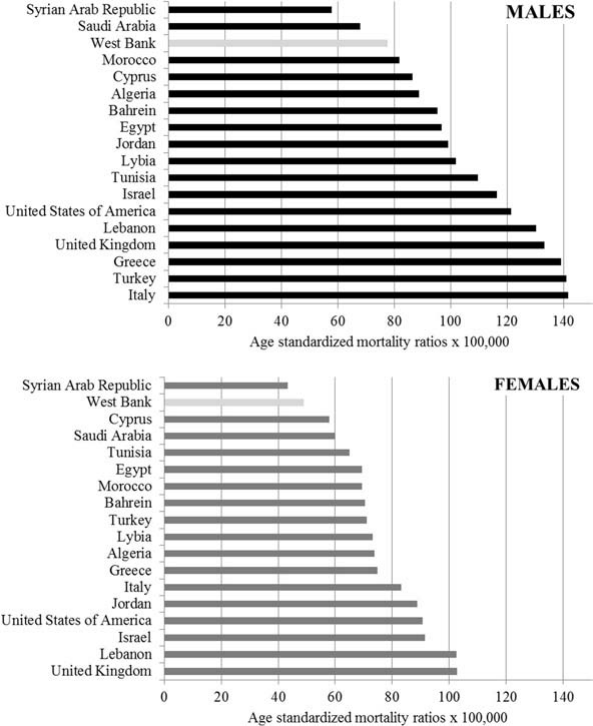
International Comparison for estimated Age-Standardised Cancer Mortality Ratios, by sex. (Source: Ferlay Jet al 2010; IARC, 2010)

The cancer burden is increasing rapidly in developing countries due to a variety of reasons. These include the increase in modifiable risk factors (smoking, ingestion of western diets and lack of exercise, environmental pressures) in addition to the increase in cancers of infectious diseases origin. Lack of access to prevention and control cancer services, late stage diagnoses and inadequate treatment facilities also lead to higher cancer mortality in these countries [2]. For the period 1999–2009, mortality rates for both males and females in the West Bank were lower than some western countries such as the United States of America, the United Kingdom and Italy, as well as some Arab countries such as Lebanon, Jordan and Egypt. The mortality rate among Palestinian males was higher than that for Saudi Arabia and Syria, while the mortality rate was higher among Palestinian females compared to Syrians (Fig. 1). It is worth noting however, that the data referred to in the figure estimates are based on fragmented data provided by national statistical services in some of the listed countries, so the results should be interpreted with caution.

Like other Arab countries, Palestine began to record increasing rates of cancer during the past few years, although these rates are still lower than neighbouring countries [3]. The crude incidence rate in 2005 was 49.2 per 100,000 in the West Bank and 32.7 per 100,000 in the Gaza Strip. The cancer mortality rate for both the West Bank and Gaza Strip was 27.8 per 100,000. The most common type of cancer among Palestinian men is lung cancer followed by prostate and colorectal cancers, while the most common among women is breast cancer followed by colorectal cancer [3, 4].

The Cancer Registry was established in Palestine in 1998 in two sites, one in the West Bank and one in the Gaza Strip, given the de facto separation of the two Palestinian regions due to the political context. Thus the physical/geographic separation of the West Bank from the Gaza Strip and the restrictions on movement in different parts of the West Bank make it difficult to have an active and effective reporting system. However, efforts continue to be made to register cases even if registration is delayed.

**Fig. 2:**
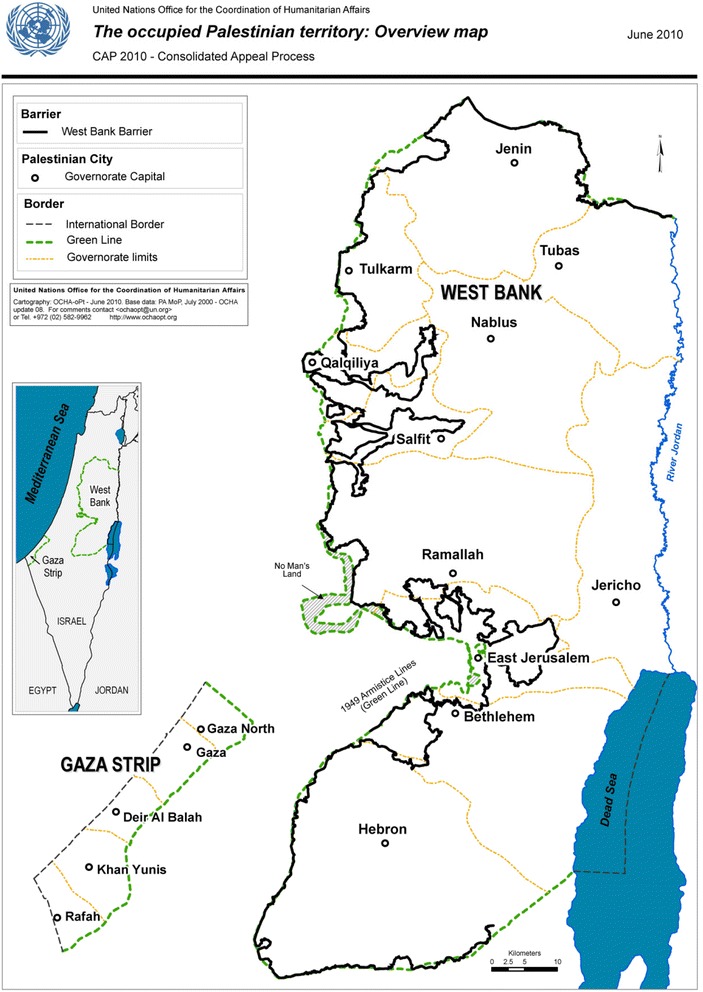
Map of West Bank governorates

Given this context, an analysis of occupied Palestinian territory mortality data is of special significance as results can be used to improve the existing registration methods as well as tools for policy and interventions [5]. Thus, this paper aims to evaluate the quality of mortality data in the occupied Palestinian territory and to assess variations in cancer mortality in different West Bank governorates (Fig. 2).

## Methods

Causes of death data were obtained from the death registry within the Palestinian Health Information Centre – Ministry of Health for the years 1999–2009. The process of mortality data collection has been described in details elsewhere [6]. The data included in this analysis are based on the underlying cause of death and coded according to the International Statistical Classification of Diseases and Related Health Problems, 10th Revision (ICD-10) [7].

The ICD-10 has been used by the Ministry of Health since the mid-1990s. Before 2003, ICD-10 sub-codes were grouped into larger groups except for selected important diseases. Valid ICD-10 codes from 1999 onwards were included in the analysis. The list of cause-related ICD-10 codes analysed is reported in Additional file 1: Table S1.

The completeness of the death registry was assessed by estimating the expected number of deaths for each year. In fact, two crude mortality rates reported by the Palestinian Central Bureau of Statistics (PCBS) (Table 1) were used. The latter rates are estimated by the PCBS based on the complete data of the census and population registry. Two mortality rates provided by the PCBS were used: mortality rates based on the 1997 census forward projections (1997 census estimates) and mortality rates based on the adjusted backward projections based on the 2007 census (2007 census estimates) [8].

**Table 1:** Estimated completeness of death registry of West Bank, 1999-2009

Year	Midyear population^a^	N. deaths observed (a)	N. deaths expected		% of completeness	
			1997 census estimates (b)	2007 census estimates (c)	Min (a) × 100/ (b)	Max (a) × 100/ (c)
1997	1,787,562	5,303	8,688	6,256	61.0	84.0
1998	1,818,114	5,303	8,600	6,327	61.7	83.8
1999	1,853,122	5,303	8,487	6,412	62.5	82.7
2000	1,892,657	5,473	8,422	6,511	65.0	84.1
2001	1,943,329	4,905	8,395	6,646	58.4	73.8
2002	2,003,852	5,598	8,436	6,813	66.4	82.2
2003	2,066,279	5,786	8,472	6,984	68.3	82.8
2004	2,130,491	5,902	8,543	7,158	69.1	82.4
2005	2,196,418	5,813	8,610	7,336	67.5	79.2
2006	2,257,682	4,996	8,692	7,496	57.5	66.7
2007	2,323,469	5,859	8,783	7,667	66.7	76.4
2008	2,385,180	6,425	8,849	7,823	72.6	82.1
2009	2,448,433	6,469	8,937	7,982	72.4	81.0

^a^Source: Palestinian Central Bureau of Statistics

Analyses were carried out for reported deaths from all of the West Bank governorates. Although Palestinian East Jerusalem is one of the eleven West Bank governorates, it was excluded from the analysis because data were available only for a small proportion of Palestinians living under Palestinian control. The majority of Palestinian East Jerusalemites are under direct Israeli control. Therefore, death certificates are not issued by the Palestinian Ministry of Health (PMoH). The data included deaths for 11 years for all governorates except Jericho, which has missing information for the year 2006.

Classical mortality indicators were computed by a Fortran package for population mortality analyses developed in the Department of Biology, in Pisa University [9].

Analyses were carried out using a Palestinian population structure obtained from the PCBS for the year 2007. To allow for international comparison, direct age standardised mortality rates were obtained using the World Population Structure [10] and reported with 95 % confidence intervals (95 % CI). Crude and standardised rates are reported per 100,000 inhabitants.

Standardised mortality ratios, defined as the ratio of observed number of deaths by the expected ones, were computed and reported as percentages (SMR%) with associated 95 % confidence intervals (95 % CI). The expected number of deaths was obtained using the total population in the studied areas, i.e. in the West Bank excluding East Jerusalem, as a reference.

In order to compare mortality patterns in urban, rural and camp areas and given the absence of detailed demographic data, standardised proportional mortality ratios were computed and reported as percentages (SPMR%). These proportional ratios were computed for specific causes. The expected number of deaths was computed using the same reference population as for the SMR. Detailed results are also reported in the supplemental material.

Almost two million people were living in the studied areas in 2007 (Table 2). The percentage distribution of the population (Additional file 1: Table S2) is typical of developing countries. 77 % of males and 75.8 % of females were below 35 years old, while 2.9 % of males and 4.1 % of females were older than 65 year. Hebron had the youngest population with 80 % of males and females below 35 years of age.

**Table 2:** All causes mortality: number of deaths, crude and standardized rates and CI95% (ref, World pop, WHO, 2002), by sex and district. West-Bank, 1999-2009

District	Population^b^			Total number of deaths^c^			Crude rates x 100,000			Direct Standardized Rates for 100,000 (ref, WHO, 2002)			
	Male	Female	Total	Male	Female	Total	Male	Female	Total	Male		Female	
										Std Rate	CI95%	Std Rate	CI95%
Jenin-Tubas	152,352	147,619	299,971	5,954	4,754	10,708	355.3	292.8	324.5	686.0	666.9 - 705.1	455.3	441.7 - 468.9
Tulkarm	79,202	77,590	156,792	3,281	2,792	6,073	376.6	327.1	352.1	757.7	730.1 - 785.3	485.2	466.7 - 503.7
Nablus	159,776	156,180	315,956	6,125	4,841	10,966	348.5	281.8	315.5	692.8	674.2 - 711.4	456.7	443.4 - 470.1
Qalqelia	45,409	44,165	89,574	1,449	1,267	2,716	290.1	260.8	275.6	659.2	621.9 - 696.4	493.9	465.2 - 522.6
Salfeet	29,884	28,916	58,800	987	819	1,806	300.3	257.5	279.2	597.6	556.8 - 638.4	409.6	380.2 - 439.0
*North*	*466,623*	*454,470*	*921,093*	*17,796*	*14,473*	*32,269*	*346.7*	*289.5*	*318.5*	*691.7*	*680.7 - 702.6*	*491.4*	*453.5 - 469.2*
Ramallah	132,375	131,057	263,432	4,448	3,420	7,868	305.5	237.2	271.5	615.0	595.8 - 634.1	346.4	334.2 - 358.6
Jericho^a^	20,227	20,176	40,403	489	342	831	219.8	154.1	187.0	547.7	492.6 - 602.7	335.6	296.7 - 374.4
*Center*	*152,602*	*151,233*	*303,835*	*4,937*	*3,762*	*8,699*	*294.1*	*226.1*	*260.3*	*610.1*	*592.0 - 628.2*	*344.8*	*333.3 - 356.4*
Bethlehem	86,550	83,416	169,966	3,011	2,469	5,480	316.3	269.1	293.1	606.7	583.7 - 629.8	439.7	421.6 - 457.8
Hebron	274,480	263,780	538,260	7,468	5,711	13,179	247.3	196.8	222.6	589.6	574.7 - 604.6	426.6	414.4 - 438.8
*South*	*361,030*	*347,196*	*708,226*	*10,479*	*8,180*	*18,659*	*263.9*	*214.2*	*239.5*	*594.8*	*582.3 - 607.4*	*432.6*	*422.5 - 442.8*
West-Bank	980,255	952,899	1,933,154	33,212	26,415	59,627	308.0	252.0	280.4	644.0	636.5 - 651.5	429.1	423.6 - 434.5

^a^Total number of deaths analysed on 10 years not eleven

^b^Source: Palestinian Central Bureau of Statistics, 2007; ^c^Source Palestinian Ministry of Health, 1999–2009

The PMoH gave the Institute of Community and Public Health authorisation to analyse the data in 2005. In addition, Birzeit University’s Central Ethics Committee stipulates, in item 5 under General Guidelines, that "Research entailing the use and /or analysis of already collected data, such as, for example, the Palestinian Central Bureau of Statistics data sets, and other such data which were collected by various institutions and researchers; or research entailing reading texts and analyzing for content, do not need ethical reviews". As such, ethical review for this study was waived, given that data were collected by others and did not reveal the identity of participants in any way.

## Results and Discussion

### Quality of mortality data

The percentages of ill-defined diseases (Table 3) decreased overtime, with some fluctuation between 2003 and 2007, indicating improvement with time in the quality of mortality data. The northern governorates had the highest percentages of ill-defined diseases compared to the central and southern governorates of the West Bank. Record completeness ranged between 70-85 %, and the percentage of ill-defined diseases ranged between 7.6 % and 12.0 %.

**Table 3:** Proportion of ill-defined diseases (ICD.10 = R00-R99) by year and governorates. West Bank, 1999-2009

Years	Ill-defined diseases		All causes	Governorates	Ill defined diseases		All causes
	N.	%	N.		N.	%	N.
1999	568	10.8	5,240	Jenin-Tubas	812	9.3	10,708
2000	544	10.1	5,394	Tulkarm	553	10.8	6,073
2001	462	9.7	4,740	Nablus	1,171	12.7	10,966
2002	502	9.3	5,423	Qalqilya	326	11.8	2,716
2003	556	9.4	5,918	Salfit	216	11.2	1,806
2004	493	8.8	5,583	*North*	*3,078*	*11.1*	*32,269*
2005	459	8.7	5,285	Ramallah	634	8.2	7,868
2006	468	8.7	5,378	Jericho^a^	82	9.0	831
2007	475	8.7	5,487	*Center*	*716*	*8.3*	*8,716*
2008	472	8.4	5,613	Bethlehemme	531	9.5	5,480
2009	391	7.0	5,583	Hebron	1,065	7.5	13,179
				*South*	*1,596*	*8.1*	*18,659*
Total	6,390^b^	9.1	59,645	West-Bank	5,390	9.0	59,627

^a^Jericho data analysed on 10 years not eleven

^b^Ill defined diseases related to cancer were less than 60 cases

Source: Palestinian Ministry of Health (PMoH), 1999–2009

### Mortality from all causes

In the period 1999–2009, a total of 59,627 deaths were analysed with a yearly average of 5,421 deaths (3,019 males and 2,401 females). The crude death rate for males was 308 and 252 for females per 100,000 per year, with a gender difference of 56 deaths per 100,000. The gender difference in the crude mortality rate was highest in the central governorates, with 68 per 100,000, and least in the southern governorates, with 49.7 per 100,000. Crude mortality rates were higher for both males and females in the northern governorates compared to the central and southern governorates. Direct age standardized mortality rates, computed to allow for international comparison, revealed higher mortality rates in the northern governorates, with the exception of the Salfit governorate. Direct standardised rates in the central and southern governorates among males were lower than in the whole of the West Bank; they were also lower in the central governorates among females, while the standardised values for the southern governorates were similar to those values obtained for the whole of the West Bank

### Cancer mortality

For cancer mortality, different patterns in the SMR distribution were observed according to both gender and governorates (Table 4). Among males the highest SMRs were found in Jenin-Tubas in the north and in Bethlehem in the south, and the lowest in Qalqilya in the north and Ramallah in the center of the West Bank. Among females, the highest SMRs were also found in Bethlehem in the south and the lowest in the central governorates of Ramallah and Jericho. The most common cause of death out of all cancer types was lung cancer among males, with 781 deaths, and breast cancer among females, with 584 deaths (Table 4). For males, the highest values of lung cancer SMRs were observed in Jenin (SMR 153.5; 95%CI: 132.6-176.8) in the north, and in Bethlehem (SMR 133.3; 95%CI: 108.8-161.7) in the south, while the SMR was lower than expected in Hebron in the south. Among females the highest lung cancer mortality was found in Bethlehem (SMR 166.4; 95%CI: 113.1-236.3) in the south. The SMR for female breast cancer was highest in the south (SMR 119.3; 95%CI: 103.9-136.2), especially in Bethlehem, and lowest in all of the centre’s governorates (SMR 76.9; 95%CI: 60.8-95.9) and in the Ramallah governorate. Prostate cancer was lowest in the centre (SMR 52.0; 95%CI: 34.5-75.1), mainly Ramallah, and similarly high in the south (SMR 118.6; 95%CI: 98.9-141.0). The genital cancer mortality pattern among females (Additional file 1: Table S3) was somewhat different from breast cancer mortality patterns: the highest SMR was found again in Bethlehem in the south but also in Qalqilya in the north. No deaths with female genital cancer were reported for Jericho in the central West Bank. No regional variation was found for liver, gallbladder, pancreas and stomach cancers for both males and females. We found high values of SMR among males in Bethlehem in the south for colon cancer and in Nablus in the north for bladder and brain cancer, while for brain cancer among females, a high SMR was found only in Bethlehem in the south.

**Table 4:** Observed, expected number of deaths, Standardized Mortality Ratio (SMR%) and CI95% for all cancers, lung, prostate and breast, by sex and West Bank districts, 1999-2009

District	Male				Female			
	Obs.	Exp.	SMR%	CI95%	Obs.	Exp.	SMR%	CI95%
All cancers (ICD.10 = C00-C99)								
Jenin-Tubas	610	549.0	111.1	102.4 - 120.2	441	445.0	99.2	90.2 - 108.9
Tulkarm	291	299.0	97.3	86.5 - 109.2	249	259.0	96.3	84.7 - 109.0
Nablus	640	604.0	105.9	97.9 - 114.5	494	486.0	101.7	93.0 - 111.1
Qalqilya	119	150.0	79.4	65.8 - 95.0	117	119.0	98.3	81.3 - 117.8
Salfit	94	109.0	85.9	69.4 - 105.1	87	88.0	99.2	79.5 - 122.4
*North*	*1,754*	*1,712.0*	*102.5*	*97.7 - 107.4*	*1,388*	*1,396.0*	*99.5*	*94.3 - 104.8*
Ramallah	437	506.0	86.4	78.5 - 94.9	337	434.0	77.6	69.6 - 86.4
Jericho	45	58.0	78.3	57.1 - 104.7	30	48.0	62.6	42.2 - 89.4
*Center*	*482*	*563.0*	*85.6*	*78.1 - 93.6*	*367*	*482.0*	*76.1*	*68.5 - 84.3*
Bethlehem	397	336.0	118.1	106.8 - 130.4	358	249.0	143.9	129.3 - 159.6
Hebron	790	812.0	97.3	90.6 - 104.3	607	594.0	102.2	94.3 - 110.7
*South*	*1,187*	*1,148.0*	*103.4*	*97.6 - 109.4*	*965*	*843.0*	*114.5*	*107.4 - 122.0*
West-Bank	3,423	3,423.0	100.0	-	2,720	2,720.0	100.0	-
Lung (ICD.10 = C34)								
Jenin-Tubas	193	126.0	153.5	132.6 - 176.8	35	34.0	104.4	72.7 - 145.2
Tulkarm	61	69.0	88.3	67.6 - 113.5	17	20.0	86.2	50.2 - 138.0
Nablus	132	140.0	94.6	79.1 - 112.2	31	37.0	84.5	57.4 - 119.9
Qalqilya	29	34.0	84.9	56.9 - 122.0	10	9.0	112.7	54.1 - 207.2
Salfit	16	25.0	64.4	36.8 - 104.6	7	*7.0*	104.6	42.0 - 215.6
*North*	*431*	*393.0*	*109.6*	*99.5 - 120.4*	*100*	106.0	*94.8*	*77.1 - 115.3*
Ramallah	108	116.0	93.0	76.3 - 112.3	35	33.0	105.6	73.5 - 146.9
Jericho	19	13.0	145.4	87.5 - 227.1	4	*4.0*	112.7	30.7 - 288.*5*
*Center*	*127*	*129.0*	*98.3*	*81.9 - 116.9*	*39*	37.0	*106.3*	*75.6 - 145.3*
Bethlehem	103	77.0	133.3	108.8 - 161.7	31	19.0	166.4	113.1 - 236.3
Hebron	120	181.0	66.2	54.9 - 79.2	34	*43.0*	78.8	54.5 - 110.1
*South*	*223*	*259.0*	*86.3*	*75.3 - 98.4*	*65*	62.0	*105.2*	*81.2 - 134.1*
West-Bank	781	781.0	100.0	-	204	204.0	100.0	-
	Prostate (ICD.10 = 61)				Breast (ICD.10 = C50)			
Jenin-Tubas	53	53.0	101.0	75.7 - 132.2	77	95.0	81.0	63.9 - 101.2
Tulkarm	28	28.0	99.0	66.0 - 143.6	56	56.0	100.9	76.2 - 131.1
Nablus	68	57.0	119.6	92.9 - 151.7	108	106.0	102.2	83.9 - 123.4
Qalqilya	10	14.0	73.6	35.3 - 135.4	21	26.0	81.0	50.1 - 123.8
Salfit	9	11.0	81.1	37.1 - 153.9	27	18.0	146.4	96.5 - 213.0
*North*	*168*	*162.0*	*103.6*	*88.5 - 120.5*	*289*	*301.0*	*96.1*	*85.4 - 107.9*
Ramallah	22	49.0	44.9	28.1 - 68.0	69	91.0	76.1	59.2 - 96.3
Jericho	6	5.0	123.1	45.1 - 268.0	9	11.0	83.5	38.2 - 158.5
*Center*	*28*	*54.0*	*52.0*	*34.5 - 75.1*	*78*	*102.0*	*76.9*	*60.8 - 95.9*
Bethlehem	41	33.0	123.9	88.9 - 168.1	83	54.0	155.1	123.5 - 192.3
Hebron	87	75.0	116.2	93.1 - 143.4	134	128.0	104.3	87.4 - 123.6
*South*	*128*	*108.0*	*118.6*	*98.9 - 141.0*	*217*	*182.0*	*119.3*	*103.9 - 136.2*
West-Bank	324	324.0	100.0	-	584	584.0	100.0	-

Source: Palestinian Ministry of Health, 1999–2009

### SPMR

SPMR% (Additional file 1: Table S4) showed similar values in the urban areas (SPMR 101.5; 95%CI: 96.8-106.2), rural areas (SPMR 99.6; 95%CI: 94.4-104.9) and camps (SPMR 90.9; 95%CI: 78.7-104.4) among males. Among females, SPMR was lowest in rural areas (SPMR 89.5; 95%CI: 84.1-95.1), highest in urban ones (SPMR 107.9; 95%CI: 102.5-113.5) but did not differ from the reference population in refugee camps (SPMR 107.8; 95%CI: 93.1-124.0).

Overall available mortality data for the West Bank of the occupied Palestinian territory is of medium quality according to the WHO’s proposed criteria [10]. The proportion of ill-defined diseases has decreased over time, reaching an average of 9 % overall and ranging between 7 % in 2009 and 11 % in 2003. This improvement in the quality of death data registration could be due to officials realising the importance of establishing good vital statistics registries in countries and territories with limited resources such as the occupied Palestinian territory. It could also be due to an observed gradual improvement in the capacities of personnel working with the PMoH and other governmental offices. Furthermore, although the level of death registry completeness was very good, differences in completeness between governorates and localities could not be assessed and are important to study in the future to ensure proper national coverage.

Regional variation in the proportion of ill-defined diseases indicates better quality of death certificates in the centre (8.3 %) and south (8.1 %) than in the north (11.1 %) of the West Bank. Thus the focus of future implementations should be on continuous training concerning the importance of accurate recording and refresher courses to support any changes in ICD classification or improvements in procedures and methodologies over time. The target audience should be medical students and medical residents and officers. It is important to emphasise that training for filling in death certificates should not be only in the form of lectures. Indeed, this is because interactive training workshops have been shown to be more effective than listening to a lecturer and receiving printed materials [11]. At the same time, local experience indicates that hands-on supportive training and supervision can also be effective. Training should target physicians working in both hospitals and in communities at the primary health care level, especially those working in rural communities [12] where follow up and supervision may be sub-optimal.

Among the northern governorates, Jenin-Tubas, Tulkarm and Nablus showed the highest total West Bank mortality for males and females. The lowest total mortality was found in all central and southern governorates, but also in Salfit in the north. Moreover the results of the study detected regional variations in the most common cancer deaths. Mortality by all neoplasms showed variations in geographic patterns, with high values only in Bethlehem in the south, which is mainly due to lung cancer in both genders and to all female cancers (breast and other female cancer). Moreover, males showed the highest mortality rates for colon cancer in Bethlehem as well. In the Jenin-Tubas governorates in the north of the West Bank, we also found high mortality for all cancers, which was mainly due to lung cancer. In Nablus in the north, we found the highest values for bladder and brain cancers among males. It is important to note that governorates in the West Bank were divided by the PCBS based on population size without taking into consideration the cultural and behavioural similarities and differences among governorates and locales. Thus, for example, Salfit is closer to the central governorates than the northern ones, yet it is classified as part of the northern governorates. In addition, even though Bethlehem is culturally different from Hebron and similar in general socio-economic and other characteristics to the central West Bank governorates, both Bethlehem and Hebron are considered part of the south West Bank.

Thus, the observed variations among governorates can be partially explained by the variation due to differences in the social and cultural environments leading to differences in ways of life as well as variations in access to services. Several hypotheses were developed to explain the influence of the context of place on health outcome. Such influences include environmental contamination, psychosocial stress, social isolation and disruption in social networks [13], [14] among other influences of place on health. In the Palestinian context, the central governorates (Ramallah and Jericho) were found to have the lowest standardised mortality rates for most cancers. This might be explained by the higher educational levels of the population in the central West Bank and the relative availability of and accessibility to health services compared to the north and the south. This is not restricted to medical services, but also includes exposure to awareness campaigns, screening programs and the availability of skilled specialists. Indeed, the central governorates, mainly Ramallah and Jerusalem (Jerusalem Areas under Palestinian Authority control- J2), have higher standards of living compared to the other governorates, ways of life which is more open to the outside world [15] and likely with higher levels of access to information related to prevention and promotion compared to the other governorates.

Furthermore, it is important to note that the central governorates are not as strictly segregated from each other by the Israeli army, and have fewer Israeli military check points compared to the northern ones [16].

Contextual and cultural factors may explain some of the regional variations, especially the low lung cancer mortality among males in the southern governorates. The southern governorates have the lowest cigarettes smoking prevalence compared to the northern governorates, which might explain the observed mortality rates [17]. In addition, the southern governorates have the highest standardised mortality rate for breast cancer. Socio-cultural barriers obstructing the access of women seeking health care may possibly explain these results. Initial investigations focusing on why Palestinian females in the West Bank do not seek breast cancer screening found that personal, cultural and environmental barriers prevent them from utilising screening services [18]. Initial evidence from a recent study of breast cancer among women in the occupied Palestinian territory indicates that women’s faith and reliance on God (*Tawwakkul*), as opposed to dependence (*Ittikal*) and fatalism are at play. This does not exclude a level of agency where women perceive treatment as their responsibility, but where socio-economic and cultural barriers may prevent them from seeking screening or treatment services [19]. However, more in-depth investigation is needed to explain the findings of this research.

Another possible explanation of this research’s findings is the increased presence of environmental risk in some West Bank governorates compared to others [20], [21]. The northern and southern governorates are in closer contact with the Israeli borders and area C locales. Area C locales are Palestinian areas but are under total Israeli military control. There are claims pertaining to Israel using these Palestinian areas as dumpsites for hazardous waste, which could also partially explain our findings. One the other hand, the northern and southern governorates contain substantial agricultural land, and the misuse of pesticides and fertilisers might be another possible environmental risk which could help explain our findings [22], [23], [24]. Overall, the results of this study highlight the need to understand why such regional variations exist. That is, more investigations need to be completed so that we are better able to understand the patterns observed in this study as well as the risk factors associated with cancer.

### Strength and limitations

This study is based on death certificates collected by the Palestinian Ministry of Health. The quality of death certificates has improved over time, but more efforts are needed, especially in training medical students, medical residence and doctors in the urban and rural communities. Some northern governorates had poorer quality data compared to other governorates. Thus, the results should be cautiously interpreted, and hopefully, this analysis will help in alerting officials and prompt them to support the northern governorate to improve their death registry. Likewise, we hope that this analysis will also bring in the realization among Palestinian policymakers that the issue of data quality and registry completeness should to be a national priority.

Some potentially important regional variations in cancer mortality were not found to be statistically significant, perhaps because of the small number of reported cases dying from certain types of cancers or because of a problem with underreporting. Nevertheless, the results of this ecological study are useful to generate hypotheses for further research in this field.

## Conclusion

This study documents regional variations in deaths due to cancer among the Palestinian West Bank population. The observed variations could be explained by personal, contextual and environmental factors that need to be investigated in-depth in order to inform policy makers and chart out further training needs and other activities, such as hands-on supervision of the death registering process, so that registries can become an adequate source of information as a tool for policies and practices.

## Additional file

Additional file 1:
**Supplemental material**. (DOCX 58 kb)

## Abbreviations

oPt: Occupied Palestinian territory
ICD 10: International Statistical Classification of Diseases and Related Health Problems, 10th Revision
PCBS: Palestinian Central Bureau of Statistics
PMoH: Palestinian Ministry of Health
SMR: Standardised Mortality Ratios
SPMR: Standardised Proportional Mortality Ratios
