# Supplementary material for: Cancer mortality in the West Bank, Occupied Palestinian Territory
Source: BMC Public Health. 2016 Jan 26;16:76. doi: 10.1186/s12889-016-2715-8 (PMC4727410; doi:10.1186/s12889-016-2715-8)
Supplement: Additional file 1: — Supplemental material. (DOCX 58 kb) [file 12889_2016_2715_MOESM1_ESM.docx]

**Supplemental material**

**Table S1: List of causes of examined death and number of deaths by sex. West Bank, 1999 - 2009**

| Cause of death | ICD.10 code |  | N. of deaths | |
| --- | --- | --- | --- | --- |
|  |  |  | Male | Female |
| All causes |  |  | 33,212 | 26,415 |
| Ill-defined diseases | R00-R99 |  | 2,554 | 2,836 |
| All cancers | C00-C99 |  | 3,423 | 2,720 |
| Stomach | C16 |  | 201 | 99 |
| Colon | C18 |  | 110 | 311 |
| Rectum | C20 |  | 41 | 33 |
| Liver and Gallbladder | C22 |  | 262 | 263 |
| Pancreas | C25 |  | 141 | 103 |
| Lung | C34 |  | 781 | 204 |
| Breast | C50 |  | 14 | 584 |
| Female Genital Organs | C51-C58,D06 |  | - | 208 |
| Prostate | C61 |  | 324 | - |
| Bladder | C67 |  | 125 | 32 |
| Brain | C71 |  | 276 | 201 |
| Lymphoma | C81-C85 |  | 160 | 103 |
| Leukaemia | C90-C95 |  | 319 | 230 |

**Table S2: Percentage distribution of the population, age, groups and districts. West Bank, 2007**

| District | Males | | | Females | | |
| --- | --- | --- | --- | --- | --- | --- |
|  | Age groups (%) | | | | | |
|  | <35 | 35-64 | 65+ | <35 | 35-64 | 65+ |
| Jenin-Tubas | 76.4 | 20.6 | 3.0 | 75.0 | 20.6 | 4.5 |
| Tulkarem | 74.8 | 22.1 | 3.1 | 72.5 | 22.5 | 5.0 |
| Nablus | 74.8 | 22.0 | 3.1 | 73.6 | 22.0 | 4.4 |
| Qalqilya | 77.5 | 19.9 | 2.6 | 77.0 | 19.4 | 3.6 |
| Salfit | 76.9 | 19.9 | 3.1 | 75.0 | 20.3 | 4.7 |
| ***North*** | ***75.7*** | ***21.2*** | ***3.0*** | ***74.3*** | ***21.3*** | ***4.5*** |
|  |  |  |  |  |  |  |
| Ramallah | 75.4 | 21.4 | 3.1 | 73.4 | 21.4 | 5.2 |
| Jericho | 76.8 | 20.8 | 2.3 | 75.9 | 20.8 | 3.3 |
| ***Center*** | ***75.6*** | ***21.3*** | ***3.0*** | ***73.7*** | ***21.3*** | ***5.0*** |
|  |  |  |  |  |  |  |
| Bethlehem | 75.3 | 21.4 | 3.3 | 74.7 | 21.0 | 4.3 |
| Hebron | 80.4 | 17.2 | 2.4 | 80.0 | 17.1 | 2.9 |
| ***South*** | ***79.2*** | ***18.2*** | ***2.6*** | ***78.7*** | ***18.1*** | ***3.2*** |
|  |  |  |  |  |  |  |
| **West-Bank** | **77.0** | **20.1** | **2.9** | **75.8** | **20.1** | **4.1** |

Source: Palestinian Central Bureau of Statistics, 2007

**Table S3: Observed, expected number of deaths, Standardized Mortality Ratio (SMR%) and CI95% for cancers types, by sex and West-Bank districts. 1999-2009**

| Districts | Male | | | | Female | | | |
| --- | --- | --- | --- | --- | --- | --- | --- | --- |
|  | Obs. | Exp. | SMR% | CI95% | Obs. | Exp. | SMR% | CI95% |
| Stomach (ICD.10= C16) | | | | | | | | |
| Jenin-Tubas | 32 | 32.0 | 99.3 | 67.9 - 140.1 | 33 | 26.0 | 127.9 | 88.0 - 179.6 |
| Tulkarm | 23 | 18.0 | 130.4 | 82.7 - 195.6 | 18 | 15.0 | 119.5 | 70.8 - 188.9 |
| Nablus | 35 | 35.0 | 99.0 | 69.0 - 137.7 | 19 | 28.0 | 68.2 | 41.0 - 106.4 |
| Qalqilya | 5 | 9.0 | 57.4 | 18.6 - 133.9 | 5 | 7.0 | 73.8 | 23.9 - 172.4 |
| Salfit | 10 | 7.0 | 153.8 | 73.8 - 282.8 | 6 | 5.0 | 117.4 | 43.1 - 255.6 |
| ***North*** | ***105*** | ***100.0*** | ***104.5*** | ***85.5 - 126.6*** | ***81*** | ***81.0*** | ***100.5*** | ***79.8 - 124.9*** |
| Ramallah | 28 | 30.0 | 93.1 | 61.9 - 134.5 | 16 | 25.0 | 63.4 | 36.2 - 102.9 |
| Jericho | 1 | 3.0 | 29.7 | 0.8 - 165.2 | 2 | 3.0 | 74.7 | 9.0 - 269.6 |
| ***Center*** | ***29*** | ***34.0*** | ***86.7*** | ***58.1 - 124.5*** | ***18*** | ***28.0*** | ***64.4*** | ***38.2 - 101.9*** |
| Bethlehem | 21 | 20.0 | 104.4 | 64.6 - 159.6 | 18 | 15.0 | 124.3 | 73.7 - 196.5 |
| Hebron | 46 | 47.0 | 97.9 | 71.6 - 130.5 | 39 | 33.0 | 118.3 | 84.1 - 161.8 |
| ***South*** | ***67*** | ***67.0*** | ***99.8*** | ***77.4 - 126.8*** | ***57*** | ***48.0*** | ***120.2*** | ***91.0 - 155.7*** |
| **West-Bank** | **201** | **201.0** | **100.0** | **-** | **99** | **99.0** | **100.0** | **-** |
| Colon (ICD.10= C18) | | | | | | | | |
| Jenin-Tubas | 48 | 49.0 | 98.8 | 72.9 - 131.0 | 54 | 51.0 | 105.8 | 79.5 - 138.0 |
| Tulkarm | 30 | 27.0 | 113.0 | 76.2 - 161.3 | 31 | 30.0 | 102.4 | 69.5 - 145.3 |
| Nablus | 54 | 54.0 | 100.6 | 75.6 - 131.2 | 68 | 56.0 | 121.5 | 94.4 - 154.1 |
| Qalqilya | 9 | 13.0 | 68.2 | 31.2 - 129.3 | 17 | 13.0 | 127.0 | 74.0 - 203.4 |
| Salfit | 3 | 10.0 | 31.1 | 6.4 - 90.9 | 6 | 10.0 | 59.4 | 21.8 - 129.3 |
| ***North*** | ***144*** | ***152.0*** | ***95.0*** | ***80.1 - 111.8*** | ***176*** | ***161.0*** | ***109.5*** | ***93.9 - 126.9*** |
| Ramallah | 46 | 45.0 | 102.0 | 74.7 - 136.1 | 34 | 52.0 | 65.8 | 45.6 - 91.9 |
| Jericho | 2 | 5.0 | 39.4 | 4.8 - 142.4 | 3 | 5.0 | 56.4 | 11.6 - 164.8 |
| ***Center*** | ***48*** | ***50.0*** | ***95.7*** | ***70.6 - 126.9*** | ***37*** | ***57.0*** | ***64.9*** | ***45.7 - 89.5*** |
| Bethlehem | 44 | 30.0 | 146.8 | 106.7 - 197.1 | 39 | 29.0 | 136.1 | 96.8 - 186.1 |
| Hebron | 66 | 70.0 | 94.0 | 72.7 - 119.6 | 59 | 65.0 | 91.3 | 69.5 - 117.8 |
| ***South*** | ***110*** | ***100.0*** | ***109.8*** | ***90.2 - 132.3*** | ***98*** | ***93.0*** | ***105.1*** | ***85.3 - 128.1*** |
| **West-Bank** | **302** | **302.0** | **100.0** | **-** | **311** | **311.0** | **100.0** | **-** |
| Rectum (ICD.10= C20) | | | | | | | | |
| Jenin-Tubas | 5 | 7.0 | 75.7 | 24.5 - 176.7 | 5 | 5.0 | 93.0 | 30.1 - 217.0 |
| Tulkarm | 3 | 4.0 | 82.9 | 17.1 - 242.3 | 2 | 3.0 | 62.0 | 7.5 - 224.0 |
| Nablus | 8 | 7.0 | 109.3 | 47.1 - 215.3 | 8 | 6.0 | 132.6 | 57.2 - 261.3 |
| Qalqilya | 1 | 2.0 | 56.0 | 1.4 - 311.7 | 2 | 1.0 | 139.5 | 16.9 - 503.5 |
| Salfit | 2 | 1.0 | 55.1 | 18.8 - 559.9 | 0 | 1.0 | 0.0 | 0.0 - 346.3 |
| ***North*** | ***19*** | ***21.0*** | ***92.1*** | ***55.5 - 143.9*** | ***17*** | ***17.0*** | ***99.2*** | ***57.8 - 158.9*** |
| Ramallah | 7 | 6.0 | 116.2 | 46.7 - 239.5 | 7 | 6.0 | 128.3 | 51.5 - 264.3 |
| Jericho | 0 | 1.0 | 0.0 | 0.0 - 527.9 | 0 | 1.0 | 0.0 | 0.0 - 641.8 |
| ***Center*** | ***7*** | ***7.0*** | ***104.1*** | ***41.8 - 214.5*** | ***7*** | ***6.0*** | ***116.1*** | ***46.6 - 239.1*** |
| Bethlehem | 5 | 4.0 | 124.5 | 40.3 - 290.5 | 4 | 3.0 | 132.2 | 36.0 - 338.5 |
| Hebron | 10 | 10.0 | 103.7 | 49.8 - 190.8 | 5 | 7.0 | 73.4 | 23.8 - 171.3 |
| ***South*** | ***15*** | ***14.0*** | ***109.8*** | ***61.5 - 181.2*** | ***9*** | ***10.0*** | ***91.5*** | ***41.9 - 173.6*** |
| **West-Bank** | **41** | **41.0** | **100.0** | **-** | **33** | **33.0** | **100.0** | **-** |
| Liver and Gallbladder (ICD.10= C22) | | | | | | | | |
| Jenin-Tubas | 48 | 42.0 | 114.2 | 84.2 - 151.4 | 37 | 43.0 | 85.7 | 60.3 - 118.1 |
| Tulkarm | 22 | 23.0 | 95.6 | 59.9 - 144.8 | 19 | 25.0 | 74.7 | 45.0 - 116.6 |
| Nablus | 33 | 47.0 | 70.6 | 48.6 - 99.2 | 45 | 47.0 | 95.3 | 69.5 - 127.6 |
| Qalqilya | 8 | 12.0 | 69.5 | 30.0 - 136.9 | 8 | 11.0 | 70.4 | 30.4 - 138.7 |
| Salfit | 9 | 8.0 | 108.8 | 49.8 - 206.5 | 10 | 9.0 | 115.7 | 55.6 - 212.9 |
| ***North*** | ***120*** | ***132.0*** | ***91.2*** | ***75.6 - 109.1*** | ***119*** | ***136.0*** | ***87.6*** | ***72.6 - 104.8*** |
| Ramallah | 42 | 39.0 | 107.9 | 77.7 - 145.8 | 42 | 43.0 | 97.1 | 70.0 - 131.2 |
| Jericho | 4 | 4.0 | 90.3 | 24.6 - 231.1 | 5 | 5.0 | 111.4 | 36.1 - 260.0 |
| ***Center*** | ***46*** | ***43.0*** | ***106.1*** | ***77.7 - 141.5*** | ***47*** | ***48.0*** | ***98.4*** | ***72.3 - 130.9*** |
| Bethlehem | 27 | 26.0 | 104.7 | 69.0 - 152.3 | 28 | 24.0 | 116.0 | 77.1 - 167.7 |
| Hebron | 69 | 61.0 | 112.6 | 87.6 - 142.5 | 69 | 55.0 | 124.8 | 97.1 - 157.9 |
| ***South*** | ***96*** | ***87.0*** | ***110.3*** | ***89.3 - 134.6*** | ***97*** | ***79.0*** | ***122.1*** | ***99.0 - 149.0*** |
| **West-Bank** | **262** | **262.0** | **100.0** | **-** | **263** | **263.0** | **100.0** | **-** |
| Pancreas (ICD.10= C25) | | | | | | | | |
| Jenin-Tubas | 21 | 23.0 | 92.4 | 57.2 - 141.2 | 25 | 17.0 | 145.1 | 93.9 - 214.2 |
| Tulkarm | 7 | 13.0 | 56.1 | 22.5 - 115.5 | 3 | 10.0 | 29.4 | 6.1 - 86.0 |
| Nablus | 27 | 25.0 | 107.0 | 70.5 - 155.6 | 16 | 19.0 | 86.6 | 49.5 - 140.6 |
| Qalqelia | 6 | 6.0 | 97.7 | 35.8 - 212.6 | 3 | 4.0 | 68.9 | 14.2 - 201.5 |
| Salfit | 6 | 5.0 | 132.6 | 48.6 - 288.7 | 3 | 3.0 | 87.0 | 18.0 - 254.4 |
| ***North*** | ***67*** | ***71.0*** | ***94.2*** | ***73.0 - 119.6*** | ***50*** | ***54.0*** | ***93.1*** | ***69.1 - 122.8*** |
| Ramallah | 22 | 21.0 | 105.0 | 65.8 - 159.0 | 10 | 18.0 | 57.2 | 27.5 - 105.2 |
| Jericho | 1 | 2.0 | 42.9 | 1.1 - 238.9 | 0 | 2.0 | 0.0 | 0.0 - 221.6 |
| ***Center*** | ***23*** | ***23.0*** | ***98.8*** | ***62.6 - 148.3*** | ***10*** | ***19.0*** | ***52.2*** | ***25.1 - 96.0*** |
| Bethlehem | 12 | 14.0 | 86.4 | 44.6 - 150.9 | 15 | 10.0 | 158.3 | 88.6 - 261.1 |
| Hebron | 39 | 33.0 | 119.2 | 84.8 - 163.0 | 28 | 21.0 | 135.4 | 90.0 - 195.7 |
| ***South*** | ***51*** | ***47.0*** | ***109.4*** | ***81.5 - 143.9*** | ***43*** | ***30.0*** | ***142.6*** | ***103.2 - 192.1*** |
| West-Bank | 141 | 141.0 | 100.0 | **-** | **103** | **103.0** | **100.0** | **-** |
| Female genital (ICD.10= C51-C58; D06) | | | | | | | | |
| Jenin-Tubas | - | - | - | - | 34 | 34.0 | 99.7 | 69.1 - 139.4 |
| Tulkarm | - | - | - | - | 15 | 20.0 | 74.7 | 41.8 - 123.2 |
| Nablus | - | - | - | - | 40 | 38.0 | 106.5 | 76.0 - 145.0 |
| Qalqilya | - | - | - | - | 16 | 9.0 | 176.6 | 101.0 - 286.8 |
| Salfit | - | - | - | - | 7 | 7.0 | 104.5 | 41.9 - 215.2 |
| ***North*** | - | - | - | - | ***112*** | ***108.0*** | ***104.2*** | ***85.8 - 125.4*** |
| Ramallah | - | - | - | - | 24 | 34.0 | 71.6 | 45.9 - 106.6 |
| Jericho | - | - | - | - | 0 | 4.0 | 0.0 | 0.0 - 100.7 |
| ***Center*** | - | - | - | - | ***24*** | ***37.0*** | ***64.6*** | ***41.4 - 96.1*** |
| Bethlehem | - | - | - | - | 37 | 19.0 | 194.2 | 136.7 - 267.7 |
| Hebron | - | - | - | - | 35 | 44.0 | 79.0 | 55.0 - 109.9 |
| ***South*** | - | - | - | - | ***72*** | ***63.0*** | ***113.7*** | ***88.9 - 143.2*** |
| **West-Bank** | - | - | - | - | **208** | **208.0** | **100.0** | **-** |
| Bladder (ICD.10= C67) | | | | | | | | |
| Jenin-Tubas | 18 | 20.0 | 89.0 | 52.7 - 140.6 | 4 | 5.0 | 74.1 | 20.2 - 189.8 |
| Tulkarm | 11 | 11.0 | 99.9 | 49.9 - 178.8 | 5 | 3.0 | 158.5 | 51.4 - 370.0 |
| Nablus | 33 | 22.0 | 147.7 | 101.7 - 207.5 | 9 | 6.0 | 157.3 | 72.0 - 289.6 |
| Qalqilya | 4 | 5.0 | 75.0 | 20.4 - 192.1 | 3 | 1.0 | 222.0 | 45.8 - 649.0 |
| Salfit | 4 | 4.0 | 98.2 | 26.7 - 251.3 | 1 | 1.0 | 93.9 | 2.4 - 523.2 |
| ***North*** | ***70*** | ***63.0*** | ***111.1*** | ***86.6 - 140.4*** | ***22*** | ***17.0*** | ***131.8*** | ***82.6 - 199.6*** |
| Ramallah | 14 | 19.0 | 75.2 | 41.1 - 126.1 | 1 | 5.0 | 18.9 | 0.5 - 105.3 |
| Jericho | 0 | 2.0 | 0.0 | 0.0 - 185.5 | 1 | 1.0 | 196.5 | 5.0 - 1,094.5 |
| ***Center*** | ***14*** | ***21.0*** | ***67.9*** | ***37.1 - 114.0*** | ***2*** | ***6.0*** | ***34.5*** | ***4.2 - 124.5*** |
| Bethlehem | 11 | 13.0 | 87.7 | 43.8 - 156.9 | 3 | 3.0 | 100.8 | 20.8 - 294.6 |
| Hebron | 30 | 29.0 | 103.9 | 70.1 - 148.4 | 5 | 7.0 | 76.5 | 24.8 - 178.5 |
| ***South*** | ***41*** | ***41.0*** | ***99.0*** | ***71.1 - 134.3*** | ***8*** | ***10.0*** | ***84.1*** | ***36.3 - 165.6*** |
| **West-Bank** | **125** | **125.0** | **100.0** | **-** | **32** | **32.0** | **100.0** | **-** |
| Brain (ICD.10= C71) | | | | | | | | |
| Jenin-Tubas | 40 | 44.0 | 90.9 | 65.0 - 123.8 | 29 | 32.0 | 89.5 | 59.9 - 128.5 |
| Tulkarm | 15 | 24.0 | 63.0 | 35.3 - 103.9 | 22 | 18.0 | 119.4 | 74.9 - 180.9 |
| Nablus | 65 | 48.0 | 134.8 | 101.1 - 171.9 | 40 | 36.0 | 112.5 | 80.4 - 153.3 |
| Qalqilya | 12 | 13.0 | 96.3 | 49.8 - 168.3 | 5 | 9.0 | 55.7 | 18.1 - 130.1 |
| Salfit | 7 | 9.0 | 81.7 | 32.8 - 168.4 | 7 | 6.0 | 109.8 | 44.1 - 226.2 |
| ***North*** | ***139*** | ***137.0*** | ***101.4*** | ***85.3 - 119.8*** | ***103*** | ***102.0*** | ***101.3*** | ***82.7 - 122.8*** |
| Ramallah | 32 | 40.0 | 80.7 | 55.2 - 114.0 | 14 | 31.0 | 45.9 | 25.1 - 77.1 |
| Jericho | 3 | 5.0 | 60.8 | 12.5 - 177.7 | 1 | 4.0 | 27.1 | 0.7 - 151.2 |
| ***Center*** | ***35*** | ***45.0*** | ***78.5*** | ***54.7 - 109.2*** | ***15*** | ***34.0*** | ***43.9*** | ***24.6 - 72.4*** |
| Bethlehem | 30 | 26.0 | 115.3 | 77.8 - 164.6 | 28 | 18.0 | 154.4 | 102.6 - 223.1 |
| Hebron | 72 | 68.0 | 105.3 | 82.4 - 132.6 | 55 | 47.0 | 117.1 | 88.2 - 152.4 |
| ***South*** | ***102*** | ***94.0*** | ***108.1*** | ***88.1 - 131.2*** | ***83*** | ***65.0*** | ***127.5*** | ***101.5 - 158.0*** |
| **West-Bank** | **276** | **276.0** | **100.0** | **-** | **201** | **201.0** | **100.0** | **-** |
| Lymphoma (ICD.10= C81-C85) | | | | | | | | |
| Jenin-Tubas | 28 | 26.0 | 109.7 | 72.9 - 158.6 | 15 | 17.0 | 90.6 | 50.7 - 149.4 |
| Tulkarm | 12 | 14.0 | 86.6 | 44.7 - 151.2 | 14 | 9.0 | 149.6 | 81.7 - 251.0 |
| Nablus | 31 | 28.0 | 110.8 | 75.3 - 157.3 | 22 | 18.0 | 122.0 | 76.5 - 184.8 |
| Qalqilya | 6 | 7.0 | 82.9 | 30.4 - 180.6 | 7 | 5.0 | 152.8 | 61.3 - 314.8 |
| Salfit | 6 | 5.0 | 119.9 | 43.9 - 260.9 | 3 | 3.0 | 92.2 | 19.0 - 269.6 |
| ***North*** | ***83*** | ***80.0*** | ***104.3*** | ***83.1 - 129.3*** | ***61*** | ***52.0*** | ***117.8*** | ***90.1 - 151.3*** |
| Ramallah | 28 | 23.0 | 120.8 | 80.3 - 174.6 | 17 | 16.0 | 108.4 | 63.1 - 173.6 |
| Jericho | 0 | 3.0 | 0.0 | 0.0 - 130.7 | 1 | 2.0 | 52.5 | 1.3 - 292.5 |
| ***Center*** | ***28*** | ***26.0*** | ***107.7*** | ***71.6 - 155.7*** | ***18*** | ***18.0*** | ***102.4*** | ***60.7 - 161.8*** |
| Bethlehem | 16 | 15.0 | 105.3 | 60.2 - 171.0 | 10 | 9.0 | 108.0 | 51.8 - 198.6 |
| Hebron | 33 | 39.0 | 84.1 | 57.9 - 118.1 | 14 | 24.0 | 57.4 | 31.4 - 96.4 |
| ***South*** | ***49*** | ***54.0*** | ***90.0*** | ***66.6 - 119.0*** | ***24*** | ***33.0*** | ***71.4*** | ***45.7 - 106.2*** |
| **West-Bank** | **160** | **160.0** | **100.0** | **-** | **103** | **103.0** | **100.0** | **-** |
| Leukaemia (ICD.10= C90-C95) | | | | | | | | |
| Jenin-Tubas | 44 | 51.0 | 86.8 | 63.1 - 116.5 | 44 | 37.0 | 118.7 | 86.2 - 159.3 |
| Tulkarm | 33 | 27.0 | 121.5 | 83.6 - 170.7 | 24 | 21.0 | 115.4 | 73.9 - 171.7 |
| Nablus | 68 | 55.0 | 123.5 | 95.9 - 156.6 | 40 | 40.0 | 101.2 | 72.3 - 137.8 |
| Qalqilya | 13 | 14.0 | 91.6 | 48.7 - 156.6 | 9 | 10.0 | 88.1 | 40.3 - 167.2 |
| Salfit | 10 | 10.0 | 97.7 | 46.9 - 179.7 | 2 | 7.0 | 27.2 | 3.3 - 98.1 |
| ***North*** | ***168*** | ***157.0*** | ***106.8*** | ***91.3 - 124.2*** | ***119*** | ***115.0*** | ***103.5*** | ***85.7 - 123.9*** |
| Ramallah | 26 | 46.0 | 56.4 | 36.9 - 82.7 | 30 | 36.0 | 84.5 | 57.0 - 120.6 |
| Jericho | 4 | 5.0 | 73.5 | 20.0 - 188.1 | 1 | 4.0 | 24.6 | 0.6 - 136.8 |
| ***Center*** | ***30*** | ***52.0*** | ***58.2*** | ***39.3 - 83.2*** | ***31*** | ***40.0*** | ***78.3*** | ***53.2 - 111.2*** |
| Bethlehem | 40 | 30.0 | 132.1 | 94.3 - 179.9 | 24 | 21.0 | 115.2 | 73.8 - 171.5 |
| Hebron | 81 | 80.0 | 101.4 | 80.5 - 126.0 | 56 | 55.0 | 102.5 | 77.4 - 133.1 |
| ***South*** | **121** | **110.0** | **109.8** | **91.1 - 131.2** | ***80*** | ***76.0*** | ***106.0*** | ***84.1 - 132.0*** |
| **West-Bank** | **319** | **319.0** | **100.0** | **-** | **230** | **230.0** | **100.0** | **-** |

Source: Palestinian Ministry of Health, 1999 - 2009

**Table S4: Observed and expected deaths, Standardized Proportional Mortality Ratio (SPMR%) and CI95% for all cancer by sex and type of locality. West-Bank, 1999-2009**

| Type of locality | Male | | | |  | Female | | | |
| --- | --- | --- | --- | --- | --- | --- | --- | --- | --- |
|  | Obs. | Exp. | SPMR% | CI95% |  | Obs. | Exp. | SPMR% | CI95% |
| Urban | 1,810 | 1,784.0 | 101.5 | 96.8 - 106.2 |  | 1,486 | 1,378.0 | 107.9 | 102.5 - 113.5 |
| Rural | 1,408 | 1,414.0 | 99.6 | 94.4 - 104.9 |  | 1,039 | 1,161.0 | 89.5 | 84.1 - 95.1 |
| Camp | 199 | 219.0 | 90.9 | 78.7 - 104.4 |  | 194 | 180.0 | 107.8 | 93.1 - 124.0 |
| **Total** | **3,417** | **3,417,0** | **100.0** | - |  | **2,719** | **2,719.0** | **100.0** | - |
